# Triggering of Erythrocyte Death by Triparanol

**DOI:** 10.3390/toxins7083359

**Published:** 2015-08-24

**Authors:** Arbace Officioso, Caterina Manna, Kousi Alzoubi, Florian Lang

**Affiliations:** 1 Department of Physiology, University of Tübingen, Gmelinstr. 5, 72076 Tuebingen, Germany; E-Mails: arbace.officioso@gmail.com (A.O.); kossai.z@gmail.com (K.A.); 2 Department of Biochemistry, Biophysics and General Pathology, School of Medicine and Surgery, Second University of Naples, Via L. De Crecchio 7, 80138 Naples, Italy; E-Mail: caterina.manna@unina2.it

**Keywords:** phosphatidylserine, cell volume, eryptosis, oxidative stress, calcium

## Abstract

The cholesterol synthesis inhibitor Triparanol has been shown to trigger apoptosis in several malignancies. Similar to the apoptosis of nucleated cells, erythrocytes may enter eryptosis, the suicidal death characterized by cell shrinkage and cell membrane scrambling with phosphatidylserine translocation to the erythrocyte surface. Triggers of eryptosis include oxidative stress which may activate erythrocytic Ca^2+^ permeable unselective cation channels with subsequent Ca^2+^ entry and increase of cytosolic Ca^2+^ activity ([Ca^2+^]_i_). The present study explored whether and how Triparanol induces eryptosis. To this end, phosphatidylserine exposure at the cell surface was estimated from annexin-*V*-binding, cell volume from forward scatter, hemolysis from hemoglobin release, [Ca^2+^]_i_ from Fluo3-fluorescence, and ROS formation from 2’,7’-dichlorodihydrofluorescein diacetate (DCFDA) dependent fluorescence. As a result, a 48 h exposure of human erythrocytes to Triparanol (20 µM) significantly increased DCFDA fluorescence and significantly increased Fluo3-fluorescence. Triparanol (15 µM) significantly increased the percentage of annexin-*V*-binding cells, and significantly decreased the forward scatter. The effect of Triparanol on annexin-*V*-binding was significantly blunted, but not abolished by removal of extracellular Ca^2+^. In conclusion, Triparanol leads to eryptosis, the suicidal erythrocyte death characterized by cell shrinkage and phospholipid scrambling of the erythrocyte cell membrane. Triparanol is at least in part effective by stimulating ROS formation and Ca^2+^ entry.

## 1. Introduction

Triparanol, a 3β-hydroxysterol-Δ24-reductase inhibitor and thus inhibitor of cholesterol synthesis [1,2,3,4], has been shown to inhibit proliferation and trigger apoptosis in several malignancies including leukemia, melanoma, chondrosarcoma, and cancer of lung, breast, liver, pancreas, or prostate [5,6,7]. Triparanol may be teratogenic [4,8] and may lead to myotonia, cataract and baldness [9].

Triparanol is partially effective by interference with the Hedgehog pathway [1,5,7,10,11,12]. Triparanol has further been shown to increase cytosolic Ca^2+^ activity ([Ca^2+^]_i_), an effect attributed to Ca^2+^ release from intracellular stores [9]. Moreover, Triparanol indirectly modifies activation of K^+^ channels [2] and decreases Na^+^/K^+^ ATPase activity [13].

Similar to apoptosis of nucleated cells, erythrocytes may enter eryptosis, the suicidal death of erythrocytes characterized by cell shrinkage [14] and phosphatidylserine translocation from the inner cell membrane leaflet to the cell surface [15]. As erythrocytes lack nuclei and mitochondria, eryptosis lacks several aspects of apoptosis, such as mitochondrial depolarization and altered gene expression [15]. However, similar to apoptotic cells, eryptotic erythrocytes are engulfed by phagocytosing cells and thus rapidly removed from circulating blood [15]. Triggers of eryptosis include oxidative stress, opening of oxidant sensitive cation channels, Ca^2+^ entry and increase of [Ca^2+^]_i_. Eryptosis is further triggered by heat stress [15], ceramide exposure [16], ATP depletion [15], and caspase activation [15,17,18]. Moreover, eryptosis is influenced by casein kinase 1α, Janus-activated kinase JAK3, protein kinase C, p38 kinase, PAK2 kinase, AMP activated kinase AMPK, cGMP-dependent protein kinase, and sorafenib/sunitinib sensitive kinases [15]. Eryptosis is triggered by a variety of xenobiotics [15,19,20,21,22,23,24,25,26,27,28,29,30,31,32,33,34,35,36,37,38,39,40,41,42,43].

The present study explored whether Triparanol triggers eryptosis. To this end, human erythrocytes from healthy volunteers were treated with Triparanol and phosphatidylserine surface abundance, cell volume, [Ca^2+^]_i_ and ROS formation determined by flow cytometry.

## 2. Results and Discussion

**Figure 1:**
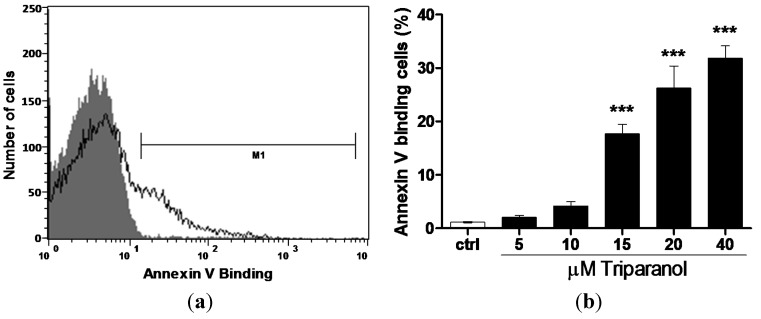
Effect of Triparanol on phosphatidylserine exposure (**a**) Original histogram of annexin-*V*-binding of erythrocytes following exposure for 48 h to Ringer solution without (grey area) and with (black line) presence of 20 µM Triparanol; (**b**) Arithmetic means ± SEM (*n* = 4) of erythrocyte annexin-*V*-binding (black bars) following incubation for 48 h to Ringer solution without or with presence of Triparanol (5–40 µM). *** (*p* < 0.001) indicate significant difference from the absence of Triparanol (ANOVA).

The present study addressed the hypothesis that Triparanol may stimulate eryptosis, the suicidal erythrocyte death characterized by cell shrinkage and cell membrane scrambling with phosphatidylserine translocation to the cell surface. In order to identify phosphatidylserine exposing erythrocytes, phosphatidylserine was quantified utilizing annexin-*V*-binding, as determined by flow cytometry (BD, Heidelberg, Germany).The erythrocytes were analyzed following incubation for 48 h in Ringer solution without or with Triparanol (5–40 µM). As illustrated in Figure 1, a 48 h exposure to Triparanol was followed by an increase of the percentage phosphatidylserine exposing erythrocytes, an effect reaching statistical significance at 15 µM Triparanol concentration.

**Figure 2:**
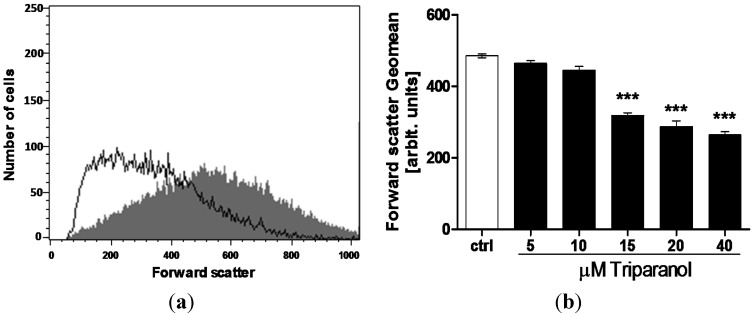
Effect of Triparanol on erythrocyte forward scatter (**a**) Original histogram of forward scatter of erythrocytes following exposure for 48 h to Ringer solution without (grey area) and with (black line) presence of 20 µM Triparanol; (**b**) Arithmetic means ± SEM (*n* = 4) of the erythrocyte forward scatter (FSC) following incubation for 48 h to Ringer solution without (white bar) or with (black bars) Triparanol (5–40 µM). *** (*p* < 0.001) indicate significant difference from the absence of Triparanol (ANOVA).

In order to quantify erythrocyte volume, forward scatter was determined utilizing flow cytometry. The measurements were again performed following a 48 h incubation in Ringer solution without or with Triparanol (5–40 µM). As shown in Figure 2, the treatment with Triparanol was followed by a decrease of erythrocyte forward scatter, an effect reaching statistical significance at 15 µM Triparanol concentration.

In order to quantify hemolysis, the hemoglobin concentration in the supernatant was determined by photometry. As a result, following a 48 h incubation the percentage of hemolytic erythrocytes was significantly (*p* < 0.01) higher following exposure to 20 µM Triparanol (2.52 ± 0.30% *n* = 8) than in the absence of Triparanol (0.10 ± 0.02% *n* = 8). The percentage of hemolytic erythrocytes remained, however, one order of magnitude lower than the percentage of annexin-*V*-binding erythrocytes.

**Figure 3:**
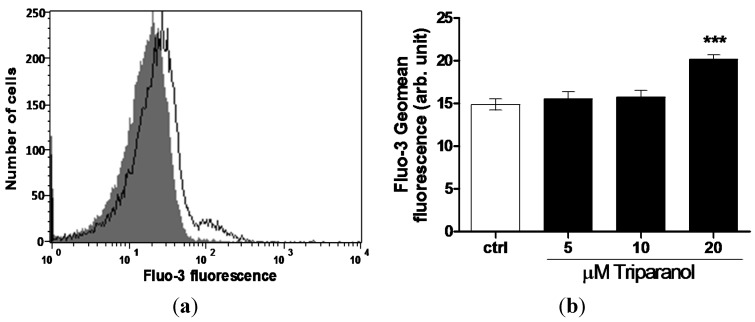
Effect of Triparanol on erythrocyte Ca^2+^ activity (**a**) Original histogram of Fluo3-fluorescence in erythrocytes following exposure for 48 h to Ringer solution without (grey area) and with (black line) presence of Triparanol (20 µM); (**b**) Arithmetic means ± SEM (*n* = 4) of the Fluo3-fluorescence (arbitrary units) in erythrocytes exposed for 48 h to Ringer solution without (white bar) or with (black bars) Triparanol (5–20 µM). *** (*p* < 0.001) indicate significant difference from the absence of Triparanol (ANOVA).

For measurement of cytosolic Ca^2+^ activity ([Ca^2+^]_i_), the erythrocytes were loaded with Fluo3 and Fluo3-fluorescence determined following a 48 h incubation in Ringer solution without or with Triparanol (5–20 µM). As shown in Figure 3, a 48 h exposure to Triparanol increased the Fluo3-fluorescence, an effect reaching statistical significance at 20 µM Triparanol.

**Figure 4:**
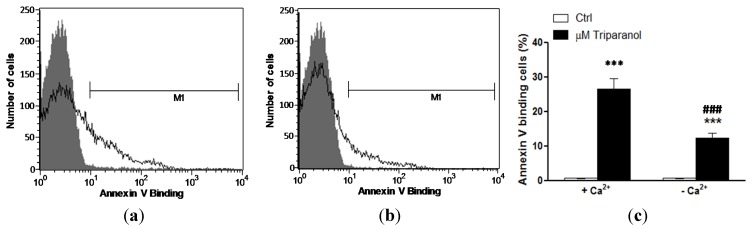
Ca^2+^ sensitivity of Triparanol-induced phosphatidylserine exposure (**a**) Original histogram of annexin-*V*-binding of erythrocytes following exposure for 48 h to Ringer solution without (grey area) and with (black line) presence of Triparanol (20 µM) in the presence; and absence (**b**) of extracellular Ca^2+^; (**c**) Arithmetic means ± SEM (*n* = 4) of annexin-*V*-binding of erythrocytes after a 48 h treatment with Ringer solution without (white bars) or with (black bars) Triparanol (20 µM) in the presence (left bars, +Ca^2+^) and absence (right bars, −Ca^2+^) of Ca^2+^. *** (*p* < 0.001) indicates significant difference from the absence of Triparanol, ^###^ (*p* < 0.001) indicate significant difference from the presence of Ca^2+^ (ANOVA).

In order to test whether Triparanol-induced translocation of phosphatidylserine required entry of extracellular Ca^2+^, erythrocytes were incubated for 48 h in the absence or presence of 20 µM Triparanol in the presence or nominal absence of extracellular Ca^2+^. As displayed in Figure 4, removal of extracellular Ca^2+^ significantly blunted the effect of Triparanol on annexin-*V*-binding. However, Triparanol significantly increased the percentage of annexin-*V*-binding erythrocytes even in the absence of extracellular Ca^2+^. Accordingly, the effect of Triparanol on cell membrane scrambling was in large part but not fully due to entry of extracellular Ca^2+^.

**Figure 5:**
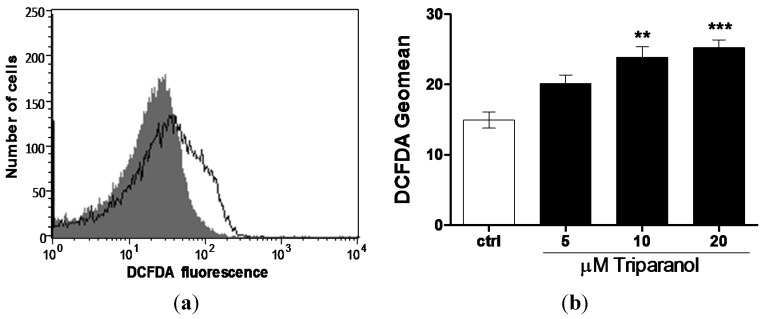
Effect of Triparanol on erythrocyte ROS formation (**a**) Original histogram of 2’,7’-dichlorodihydrofluorescein diacetate (DCFDA) fluorescence in erythrocytes following exposure for 48 h to Ringer solution without (grey area) and with (black line) presence of Triparanol (20 µM); (**b**) Arithmetic means ± SEM (*n* = 4) of the DCFDA fluorescence (arbitrary units) in erythrocytes exposed for 48 h to Ringer solution without (white bar) or with (black bars) Triparanol (5–20 µM). ** (*p* < 0.01), *** (*p* < 0.001) indicate significant difference from the absence of Triparanol (ANOVA).

Since Ca^2+^ entry and subsequent eryptosis could have been triggered by oxidative stress, reactive oxygen species (ROS) was determined utilizing 2’,7’-dichlorodihydrofluorescein diacetate (DCFDA). As illustrated in Figure 5, a 48 h exposure to Triparanol increased the DCFDA fluorescence, an effect reaching statistical significance at 10 µM Triparanol.

The concentration of reduced glutathione (GSH) was significantly higher in untreated (223 ± 13 *n* = 4) than in Triparanol (20 µM) treated (96 ± 12, *n* = 4) erythrocytes.

To test whether Triparanol is similarly effective in other enucleated cells, blood platelets were exposed to Triparanol (20 µM) for 30 min. As a result, the percentage annexin-*V*-binding blood platelets was significantly higher in Triparanol (20 µM) treated (50 ± 13, *n* = 5) than in untreated (0.5 ± 0.1, *n* = 5) blood platelets.

The present study reveals a novel effect of Triparanol, *i.e.* the triggering of suicidal erythrocyte death or eryptosis. Triparanol treatment for 48 h was followed by cell shrinkage and cell membrane scrambling with phosphatidylserine translocation to the erythrocyte surface. The concentrations required for the stimulation of eryptosis were within the range expected following the administration of 200 mg in humans [44].

The present observations were made in erythrocytes drawn from healthy individuals. The rate of eryptosis may be higher in clinical conditions with enhanced susceptibility to triggers of eryptosis, such as dehydration [32], hyperphosphatemia [42] chronic kidney disease (CKD) [24,45,46,47], hemolyticuremic syndrome [48], diabetes [49], hepatic failure [50], malignancy [15], sepsis [51], sickle-cell disease [15], beta-thalassemia [15], Hb-C and G6PD-deficiency [15], or Wilsons disease [52]. In those erythrocytes, lower Triparanol concentrations may be required for the triggering of eryptosis.

As Triparanol was added to isolated erythrocytes *in vitro*, the effect on erythrocytes was presumably due to mechanisms other than inhibition of cholesterol synthesis. The effect of Triparanol on cell membrane scrambling and cell shrinkage was paralleled by an increase of cytosolic Ca^2+^ activity ([Ca^2+^]_i_). Moreover, the effect of Triparanol on cell membrane scrambling was significantly blunted in the absence of extracellular Ca^2+^. Thus, the effect was in large part due to Ca^2+^ entry. An increase of [Ca^2+^]_i_ has previously been shown to trigger cell membrane scrambling by activating an ill-defined scramblase [15]. An increase of [Ca^2+^]_i_ has further been shown to cause erythrocyte shrinkage by activation of Ca^2+^ sensitive K^+^ channels with subsequent K^+^ exit, cell membrane hyperpolarization, Cl^−^ exit and thus cellular loss of KCl with water [14].

The stimulation of Ca^2+^ entry with subsequent increase of [Ca^2+^]_i_ following Triparanol treatment was paralleled by and at least in part due to triggering of oxidative stress, which has previously been shown to activate Ca^2+^ permeable cation channels with subsequent stimulation of Ca^2+^ entry and increase of [Ca^2+^]_i_ [15].

The physiological purpose of eryptosis is the clearance of defective erythrocytes from circulating blood prior to hemolysis [15]. Eryptosis thus serves to prevent release of hemoglobin, which would be filtered in renal glomerula, precipitate in the acidic lumen of renal tubules and thus occlude nephrons [53]. Eryptosis further accomplishes clearance of erythrocytes infected with the malaria pathogen *Plasmodium*. The pathogen imposes oxidative stress on the host erythrocyte thus leading to opening of Ca^2+^-permeable erythrocyte cation channels [15,54]. Sickle-cell trait, beta-thalassemia-trait, Hb-C and G6PD-deficiency accelerate eryptosis and subsequent clearance of infected erythrocytes, thus decreasing parasitemia and protecting against a severe course of malaria [15,55,56,57]. Accelerated eryptosis in iron deficiency [58], and following treatment with lead [58], chlorpromazine [59] or NO synthase inhibitors [59] similarly counteracts development of parasitemia. Possibly, Triparanol similarly enhances the susceptibility of *plasmodium* infected erythrocytes to eryptosis.

Stimulation of eryptosis may, however, lead to anemia, if the erythrocyte loss is not counterbalanced by an equivalent increase of erythropoiesis [15]. Moreover, phosphatdylserine exposing erythrocytes may adhere to the vascular wall [60], stimulate blood clotting and trigger thrombosis [61,62,63], thus impairing microcirculation [16,61,64,65,66,67].

## 3. Experimental Section 

### 3.1. Erythrocytes, Platelets, Solutions and Chemicals

Fresh Li-Heparin-anticoagulated blood samples were kindly provided by the blood bank of the University of Tübingen and were drawn from healthy individuals. The study is approved by the ethics committee of the University of Tübingen (184/2003 V). The blood was centrifuged at 120 *g* for 20 min at 21 °C and the platelets and leukocytes-containing supernatant was disposed. Erythrocytes were incubated *in vitro* at a hematocrit of 0.4% in Ringer solution containing (in mM) 125 NaCl, 5 KCl, 1 MgSO4, 32 *N*-2-hydroxyethylpiperazine-*N*-2-ethanesulfonic acid (HEPES; pH 7.4), 5 glucose, 1 CaCl2, at 37 °C for 24 h. Where indicated, erythrocytes were exposed to Triparanol (Sigma Aldrich, Hamburg, Germany) at the indicated concentrations.

In one series of experiments blood platelets were isolated from wild type mice. The mice were anesthetized and blood was drawn from the retroorbital plexus into tubes with 300 µL acid-citrate-dextrose buffer. Platelet rich plasma (PRP) was obtained by centrifugation at 260 *g* for 5 min. Afterwards PRP was centrifuged at 640 *g* for 5 min to pellet the platelets. After two washing steps the pellet of washed platelets was resuspended in modified Tyrode-HEPES buffer (pH 7.4, supplemented with 1 mM CaCl_2_ (Sigma Aldrich, Hamburg, Germany). All animal experiments were conducted according to the German law for the welfare of animals and were approved by local authorities.

### 3.2. Annexin-V-binding and Forward Scatter 

After incubation under the respective experimental condition, 150 µL cell suspension was washed in Ringer solution containing 5 mM CaCl_2_ and then stained with Annexin-V-FITC (1:200 dilution; ImmunoTools, Friesoythe, Germany) in this solution at 37 °C for 20 min under protection from light. The annexin V abundance at the erythrocyte surface was subsequently determined on a FACS Calibur (BD, Heidelberg, Germany). A dot plot of forward scatter (FSC) *vs.* side scatter (SSC) was set to linear scale for both parameters. The threshold of forward scatter was set at the default value of “52”.

### 3.3. Hemolysis 

For the determination of hemolysis, the samples were centrifuged (3 min at 1600 rpm, room temperature) after incubation under the respective experimental conditions and the supernatants were harvested. As a measure of hemolysis, the hemoglobin (Hb) concentration of the supernatant was determined photometrically at 405 nm. The absorption of the supernatant of erythrocytes lysed in distilled water was defined as 100% hemolysis.

### 3.4. Intracellular Ca^2+^

After incubation, erythrocytes were washed in Ringer solution and then loaded with Fluo-3/AM (Biotium, CA, USA) in Ringer solution containing 5 mM CaCl_2_ and 5 µM Fluo-3/AM. The cells were incubated at 37 °C for 30 min and washed once in Ringer solution containing 5 mM CaCl_2_. The Fluo-3/AM-loaded erythrocytes were resuspended in 200 µL Ringer. Then, Ca^2+^-dependent fluorescence intensity was measured with an excitation wavelength of 488 nm and an emission wavelength of 530 nm on a FACS Calibur.

### 3.5. Reactive Oxidant Species (ROS) 

Oxidative stress was determined utilizing 2’,7’-dichlorodihydrofluorescein diacetate (DCFDA). After incubation, a 100 µL suspension of erythrocytes was washed in Ringer solution and then stained with DCFDA (Sigma, Schnelldorf, Germany) in PBS containing DCFDA at a final concentration of 10 µM. Erythrocytes were incubated at 37 °C for 30 min in the dark and then washed in PBS. The DCFDA-loaded erythrocytes were resuspended in 200 µL Ringer solution, and ROS-dependent fluorescence intensity was measured at an excitation wavelength of 488 nm and an emission wavelength of 530 nm on a FACS Calibur (BD).

### 3.6. GSH Abundance

Reduced glutathione (GSH) abundance was determined utilizing 5-chloromethylfluorescein diacetate (5-CMFDA). After incubation, a 100 µL suspension of erythrocytes were centrifuged at 1600 rpm for 3 min at 22 °C, supernatant was discarded and cells were then stained with 5-CMFDA (Santa Cruz Biotechnology, Dallas, TX, USA) in PBS containing 5-CMFDA at a final concentration of 1 µM. Erythrocytes were incubated at 37 °C for 45 min in the dark and then washed in PBS. The 5-CMFDA-loaded erythrocytes were resuspended in 200 µL PBS, and 5-CMFDA-dependent fluorescence intensity was measured at an excitation wavelength of 488 nm and an emission wavelength of 530 nm on a FACS Calibur (BD).

### 3.7. Statistics

Data are expressed as arithmetic means ± SEM. As indicated in the figure legends, statistical analysis was made using ANOVA with Tukey’s test as post-test and t test as appropriate. *n* denotes the number of different erythrocyte specimens studied. Some variability is observed between erythrocytes drawn from different individuals. Thus, comparisons have always been made between erythrocytes from the same individual.

## 4. Conclusions

Triparanol triggers eryptosis with cell shrinkage and cell membrane scrambling, an effect paralleled by and in part due to induction of oxidative stress and increase of cytosolic Ca^2+^ activity.
